# Clinical Presentation and Long‐Term Survival Outcomes of Patients With Monoclonal Gammopathy of Renal Significance (MGRS): A Multicenter Retrospective Study

**DOI:** 10.1002/cam4.70266

**Published:** 2024-11-25

**Authors:** K. Mancuso, R. Mina, S. Rocchi, E. Antonioli, M. T. Petrucci, F. Fazio, A. Gozzetti, M. Salvini, C. S. Cartia, G. Bertuglia, F. Patriarca, A. B. Dalla Palma, S. Barbato, G. De Cicco, F. Bigi, E. Favero, P. Tacchetti, L. Pantani, I. Rizzello, M. Puppi, M. Talarico, V. Solli, A. Kanapari, M. Cavo, E. Zamagni

**Affiliations:** ^1^ IRCCS Azienda Ospedaliero‐Universitaria di Bologna Istituto di Ematologia “Seràgnoli” Bologna Italy; ^2^ Dipartimento di Scienze Mediche e Chirurgiche Università di Bologna Bologna Italy; ^3^ Myeloma Unit, Division of Hematology University of Turin and Azienda Ospedaliero‐Universitaria (AOU) Città della Salute e della Scienza di Torino Torino Italy; ^4^ Hematology Department Careggi Hospital Florence Italy; ^5^ Hematology–Azienda Policlinico Umberto I‐Department of Translational and Precision Medicine–Sapienza, University of Rome Roma Italy; ^6^ Department of Medical Science, Surgery and Neuroscience, Hematology University of Siena Siena Italy; ^7^ UOC Ematologia, ASST Sette Laghi Ospedale di Circolo e Fondazione Macchi Varese Italy; ^8^ Division of Hematology Fondazione IRCCS Policlinico San Matteo Pavia Italy; ^9^ Clinica Ematologica, Azienda Sanitaria Universitaria Friuli Centrale, Dipartimento di Medicina Università di Udine Udine Italy; ^10^ Hematology and BMT Unit Azienda Ospedaliero‐Universitaria di Parma Parma Italy

## Abstract

**Introduction:**

MGRS are new rare clinical entities, whose recognition and optimal management is evolving.

**Methods:**

To implement real‐life data, we retrospectively analysed a multicentre cohort of 60 patients with renal biopsy‐proven MGRS receiving mainly novel treatments (between 2006 and 2021) in eight Italian centres. Based on renal biopsy, patients were divided into two subgroups: AL amyloidosis (70%, *n* = 42) and other‐MGRS (30%, *n* = 18).

**Results:**

Baseline characteristics follow typical manifestations of MGRS disorders in terms of small clonal burden, laboratory and clinical features. More patients with AL amyloidosis had monotypic lambda light‐chain disease, estimated glomerular filtration rate (eGFR) ≥ 60 mL/min and nephrotic proteinuria than other‐MGRS group. The most widely used drug was bortezomib, and about one‐third of patients underwent ASCT. Overall response rate was 86% with no differences in the two subgroups. However, high‐quality hematologic responses ≥very good partial response (VGPR) were greater in AL amyloidosis than in other‐MGRS group (67% vs 28%, *p* = 0.015). The depth of haematological response influenced renal response, obtained in 32 (59%) of evaluable patients, similarly in the subgroups. Indeed, 75% patients with ≥ VGPR (*p* = 0.049) and none with stable disease (*p* ≤ 0.001) obtained a renal response. No association between renal response and histotypes (*p* = 0.9) or type of first‐line therapy (*p* = 0.3) was found. At a median follow‐up of 54.4 months (IQR 24.8–102.8), median progression‐free survival (PFS) was 100.1 months (95% CI 34.9–NR), and median overall survival not reached (95% CI 129.8–NR). No significant difference emerged between the two groups in terms of survival outcomes. Achieving ≥ VGPR was confirmed as the main independent predictor of prolonged PFS in the general population (HR = 0.29, *p* = 0.023) and AL amyloidosis group (HR 0.23; *p* = 0.023). Preserved renal function at diagnosis was predictive of improved PFS in the AL amyloidosis group (eGFR ≥ 60 mL/min: HR = 0.003; *p* = 0.018; eGFR 30–60 mL/min: HR = 0.04, *p* = 0.046).

**Conclusion:**

Further research is warranted to develop standardised response criteria and treatment strategies to improve MGRS management.

## 1 Introduction

**FIGURE 1:**
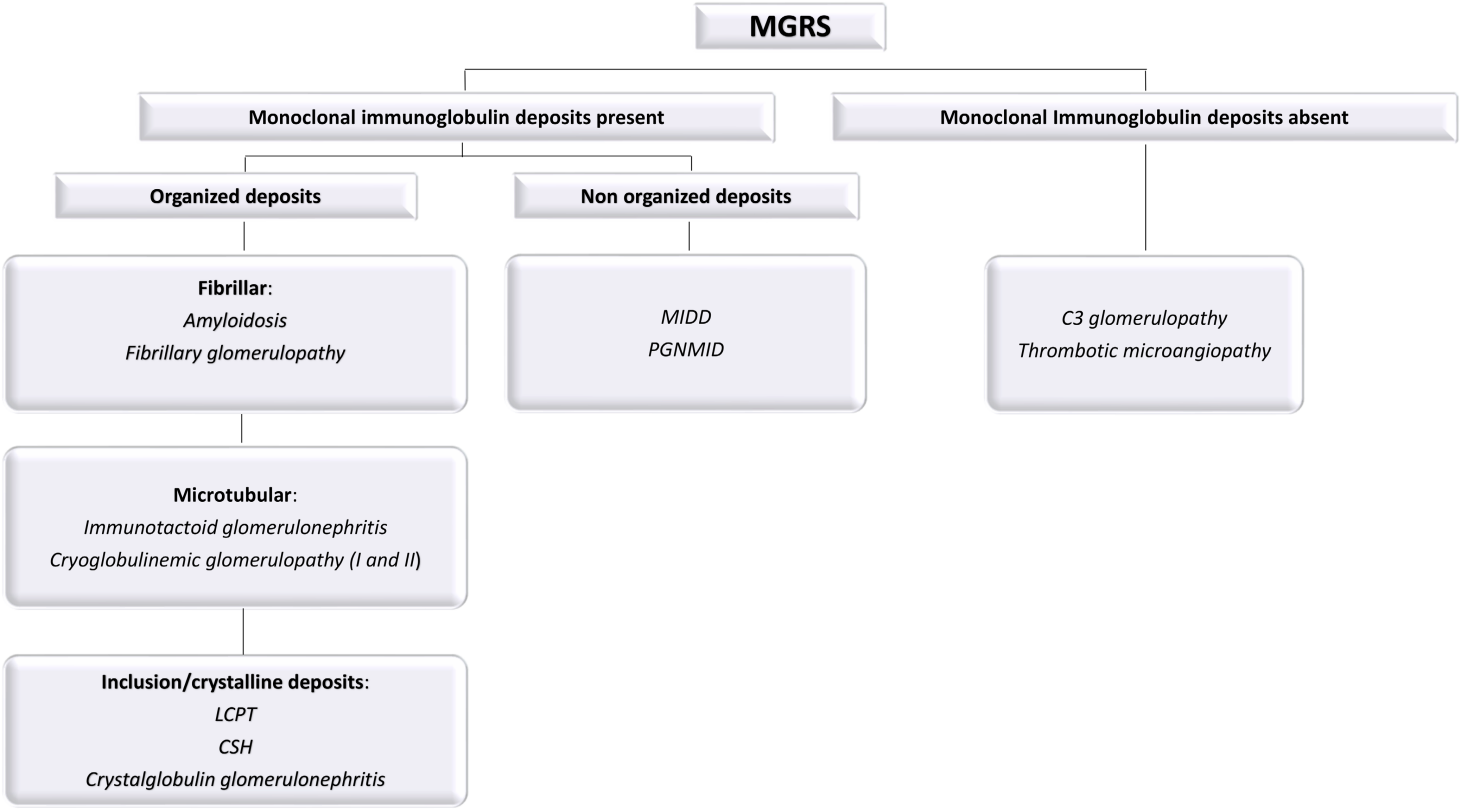
Histopathologic classification of MGRS. CSH, crystal storing histiocytosis; LCPT, light‐chain proximal tubulopathy; MIDD, monoclonal immunoglobulin deposition disease; PGNMID, proliferative glomerulonephritis and monoclonal immunoglobulin deposits (Adapted from [8]).

Monoclonal gammopathies include a broad spectrum of conditions characterized by plasma cell‐ or lymphoproliferative disorder, ranging from premalignant benign conditions (monoclonal gammopathy of undetermined significance, MGUS) to neoplastic diseases, including multiple myeloma (MM), Waldenström macroglobulinemia (WM), and chronic lymphocytic leukemia (CLL) [1, 2, 3, 4]. Kidney disease is a frequent complication of monoclonal gammopathies that manifests with a wide range of renal lesions [5, 6]. The growing recognition of the relationship between monoclonal gammopathies in the absence of symptomatic MM, WM, or CLL and kidney disease led to the need for a more accurate classification of these disorders, which were previously often misdiagnosed and without access to potentially effective medications. In an attempt to address this need, in 2012, the International Kidney and Monoclonal Gammopathy Research Group (IKMG) first introduced the term “monoclonal gammopathy of renal significance” (MGRS) to distinguish these monoclonal gammopathies from MGUS, filling the diagnostic gap without changing the definition of a malignant process [7]. Only more recently MGRS have been included in the 2022 WHO‐HAEM5 classification, referring to any clonal B‐cell or plasma‐cell (PC) disorder that does not meet current criteria for immediate treatment but produces a nephrotoxic monoclonal immunoglobulin that directly or indirectly results in kidney disease or injury [8, 9, 10]. Renal impairment is caused, by different mechanisms involving different compartments of the kidney, by the structural and physicochemical characteristics of monoclonal immunoglobulin or its fragments, most commonly secreted by a “dangerous small” clone with a low‐grade of cellular proliferation [8, 11, 12, 13, 14, 15, 16, 17]. MGRS‐related kidney lesions are classified according to the characteristics of the monoclonal immunoglobulin deposits by electron microscopy: organized, non‐organized, or absent deposits (for further details see Figure 1) [8, 9].

Due to the wide spectrum of clinical manifestations, as well as the relatively common finding of a serum paraprotein, the diagnosis of these diseases can be challenging and requires a demonstration of the link between MGRS‐related kidney lesions and the clone‐secreting pathogenic monoclonal immunoglobulin. Thus, the diagnostic algorithm is based on the complementary use of essential renal biopsy with a specific hematologic workup including serum and urine laboratory tests and bone marrow (BM) or lymph node biopsy [8, 18]. Early recognition of MGRS and subsequent timely use of therapeutic approaches, aimed at eradication of the malignant clone, are crucial to prevent potential progressive renal failure to its final stage and to improve patient outcomes [17, 19, 20, 21, 22, 23, 24, 25].

Nonetheless, as clinical trials are rare for MGRS, except for immunoglobulin light‐chain amyloidosis, data on the management of MGRS are still lacking, and there is no consensus regarding the best treatment options in the first‐line setting, and even more so in case of relapse.

A recently published real‐world survey added valuable information on the prognostic indicators and treatment outcomes of MGRS [26]. Aiming to enlarge data on the real‐life setting, we conducted a multicenter, national, retrospective study of biopsy‐proven MGRS patients, with a longer period of observation, focusing on treatment options and their efficacy, safety, and management of emerging toxicities in daily clinical practice.

## 2 Materials and Methods

This non‐interventional, observational, retrospective study was run in 8 Italian hematologic centers, with the primary purpose of evaluating the prognostic characteristics and treatment outcomes of patients with MGRS, in terms of hematological response, renal response rate, progression‐free survival (PFS), and overall survival (OS). Additional endpoints of the study included the description of patient characteristics at baseline, the most frequently used therapies, the relationship between hematological and renal responses, the impact of different MGRS histotypes and first‐line therapies on the achievement of response, and, finally, the assessment of safety and tolerability of different therapeutic strategies.

Eligible patients were aged ≥ 18 years and had a histologically documented diagnosis of MGRS according to the consensus statement of the IKMG [8]: specifically, a kidney biopsy integrated by light microscopy, immunohistochemistry (immunofluorescence), and transmission electron microscopy evaluations. MGRS kidney lesions were classified according to the 2018 consensus by the IKMG Research Group [8]. Key exclusion criteria were: monoclonal gammopathy of clinical significance (MGCS) without renal involvement, presence of any defining features of an overt lymphoplasmacytic malignancy and any myeloma‐defining event, and/or histological documentation of light‐chain cast nephropathy, AL amyloidosis with multi‐organ involvement documented by instrumental and/or histological examination.

Clinical and laboratory data were collected from the electronic medical record system. The following baseline parameters were evaluated: age, performance status with the Eastern Cooperative Oncology Group (ECOG), blood cell count, renal function (CKD‐EPI calculation), liver function, beta‐2 microglobulin, serum calcium, lactic dehydrogenase (LDH), serum albumin, serum and urine immunofixation combined with protein electrophoresis, 24‐h proteinuria, serum free light chain (sFLC) assay using the Freelite assay test (The Binding Site, Birmingham, United Kingdom), plasma cell percentage (documented at the BM biopsy), and high‐risk cytogenetic abnormalities (HRCA), including del(17p), *t*(4;14) and/or *t*(14;16) detected by fluorescence in situ hybridization (FISH). Renal impairment was defined as estimated glomerular filtration rate (eGFR) ≤ 60 mL/min.

Therapy was selected at physician's discretion, based on the patient's age, comorbidities, and characteristics of the underlying disease. The hematological response was assessed according to the International Myeloma Working Group (IMWG) consensus criteria and defined as follows: complete response (CR), negative immunofixation on the serum and urine, and < 5% plasma cells in BM aspirates; stringent complete response (sCR), CR plus normal FLC ratio and absence of clonal cells in BM biopsy by immunohistochemistry; very good partial response (VGPR), serum and urine M‐protein detectable by immunofixation but not on electrophoresis or ≥ 90% reduction in serum M‐protein plus urine M‐protein level < 100 mg per 24 h; partial response (PR), ≥ 50% reduction of serum M‐protein plus reduction in 24 h urinary M‐protein by ≥ 90% or to < 200 mg per 24 h; if the serum and urine M‐protein is unmeasurable, a ≥ 50% decrease in the difference between involved and uninvolved FLC levels is required in place of the M‐protein criteria; stable disease (SD), not meeting criteria for CR, VGPR, PR, or progressive disease (PD) [27]. Renal response was evaluated based on proteinuria and eGFR, following international recommendations for amyloidosis and defined as a reduction of > 30% in 24‐h proteinuria (in the absence of renal progression, defined by a progressive decrease of > 25% in eGFR) [28]. As AL amyloidosis is the most common pathologic phenotype of MGRS lesions, we performed a comparison between AL amyloidosis and other‐MGRS lesions.

PFS, defined as the time from the initiation of treatment to the occurrence of hematological disease progression or death, was used to assess treatment efficacy.

The safety assessment was based on reports of hematological and non‐hematological toxicity, including neutropenia, thrombocytopenia, anemia, peripheral polyneuropathy (PNP), infections, skin rash, gastro‐enteric toxicity, cardiovascular adverse events (AEs), and metabolic disease. All AEs were recorded using the common terminology criteria for adverse events (CTCAEs) version 5.0.

The study was approved by Comitato Etico Area Vasta Emilia Romagna (CE‐AVEC) and by local Ethics Committees at each participating site and was conducted in accordance with the International Conference on Harmonization Guidelines on Good Clinical Practice and the principles of the Declaration of Helsinki. All participants signed an informed consent form prior to their inclusion in the study.

### 2.1 Statistical Analysis

For descriptive analysis, dichotomous data, expressed in terms of presence or absence, and categorical data, having more than two levels, were described by absolute frequencies (*N*) and percentages (%). For numerical data, median values and interquartile range (IQR) were applied. The association among categorical variables was tested with Pearson's chi‐squared test and Fisher's exact test, as appropriate, and the differences among continuous variables among groups were tested by Wilcoxon's rank sum test. Univariate survival analyses were performed by Kaplan Meier curves and log‐rank test and by fitting the Cox proportional hazards model to calculate hazard ratios (HRs). The latter was also used for multivariate survival analyses, including only significant univariate variables. Median follow‐up was computed with the reverse Kaplan–Meier estimator, which reverses the censor and event indicator of the standard method. Inference was performed with a 95% confidence level. All analyses were performed with R version 4.2.1.

## 3 Results

### 3.1 Baseline Characteristics of Patients

From June 2006 to April 2021, 60 patients with MGRS confirmed by renal biopsy and meeting the inclusion criteria were included in this retrospective study. For the purposes of analysis, patients were divided into 2 subgroups according to the histopathological features of renal biopsy: AL amyloidosis (70%, *n* = 42) and other‐MGRS (30%, *n* = 18). The main baseline characteristics of the overall population and each subgroup at study entry are summarized in Table 1. In detail, the median age was 65 years (IQR: 55–72), with no difference between the two subgroups (*p* = not significant [ns]). There was a higher prevalence of male sex in the other‐MGRS (*n* = 15, 83%) than in the AL amyloidosis (*n* = 20, 48%, *p* = 0.010) group. IgG heavy chains were the most common isotype (*n* = 25, 48%), followed by light chains only disease (either kappa or lambda) (*n* = 18, 35%). More patients (*n* = 29, 78%) in the AL amyloidosis group had a lambda monotypic light‐chain restriction, with a higher median value (56 mg/L [IQR 21–135]) compared with patients in the other‐MGRS group (*n* = 5, 33%, *p* = 0.002, and 24 mg/L [IQR 13–36], *p* = 0.027, respectively).

**TABLE 1:** Baseline demographics and disease patient characteristics in the overall population and by histologic subtype.

Characteristics	*N*	Overall	AL amyloidosis	Other‐MGRS	*p* value
Patients, *N* (%)	60	60 (100)	42 (70)	18 (30)	
Median age (IQR), years	60	65 (55–72)	65 (57–72)	64 (52–72)	ns
Male sex, *N* (%)	60	35 (58)	20 (48)	15 (83)	0.010
Isotype, *N* (%)	52				ns
IgG *κ*/*λ*		25 (48)	16 (43)	9 (60)	ns
IgA *κ*/*λ*		5 (10)	3 (8)	2 (13)	ns
IgM *κ*/*λ*		3 (6)	2 (6)	1 (7)	ns
Light chains *k* or *λ* only		19 (36)	16 (43)	3 (20)	ns
Light chains	52				
*k*		18 (35)	8 (22)	10 (67)	0.002
*λ*		34 (65)	29 (78)	5 (33)	0.002
Serum M‐protein, median (IQR) mg/dL	43	40 (0–323)	0 (0–211)	202 (0–514)	ns
sFLC, median (IQR) mg/L	60				
*κ*		19 (10–64)	12 (8–26)	55 (23–422)	< 0.001
*λ*		34 (18–77)	56 (21–135)	24 (13–36)	0.027
*k*/*λ* ratio		0 (0–4)	0 (0–1)	3 (1–23)	< 0.001
sFLC, median (IQR) mg/L	60				
Involved		83 (38–333)	75 (40–280)	96 (37–590)	ns
Uninvolved		13 (9–20)	11 (8–18)	19 (11–26)	0.039
Type of renal lesion, *N* (%)	60				< 0.001
AL amyloidosis		42 (70)	42 (100)	0 (0)	
MIDD		7 (12)	0 (0)	7 (39)	
LCPT		5 (8)	0 (0)	5 (28)	
PGNMID		2 (3.3)	0 (0)	2 (11)	
Monoclonal FGN		2 (3.3)	0 (0)	2 (11)	
C3G with MG		1 (1.7)	0 (0)	1 (5.5)	
Cryo‐GN		1 (1.7)	0 (0)	1 (5.5)	
BM involvement (% PCs), median (IQR)	47	8 (5–10)	5 (5–10)	8 (5–10)	ns
Albumin, median (IQR) mg/dL	57	3.20 (2.7–4.1)	2.95 (2.4–3.4)	4.12 (3.6–4.5)	< 0.001
B‐2‐microglobulin, median (IQR) mg/L	50	3.3 (2.2–4.6)	2.9 (2.1–4.0)	4.6 (3.1–7.6)	0.021
Serum creatinine, median (IQR) mg/dL	57	1.2 (0.8–2.0)	1.00 (0.7–1.3)	2.3 (1.4–3.5)	< 0.001
eGFR, median (IQR) ml/min per 1.73 m^2^	56	58 (30–90)	78 (47–95)	31 (16–53)	0.001
≥ 60 mL/min, *N* (%)		28 (50)	25 (63)	3 (19)	0.003
30–60 mL/min, *N* (%)		13 (23)	8 (20)	5 (31)	*ns*
< 30 mL/min, *N* (%)		15 (27)	7 (18)	8 (50)	0.020
24‐h urine protein, median (IQR) g	57	4.0 (2.2–6.0)	4.5 (3.1–7.6)	2.4 (0.8–4.3)	0.003
Dialysis, *N* (%)	60	12 (21)	10 (25)	2 (13)	*ns*

*Note:* FISH cytogenetic analysis was performed in 10 patients, and none had high‐risk abnormalities, including del(17p), *t*(4;14), and/or *t*(14;16).

Abbreviations: BM, bone marrow; C3G, complement 3 glomerulopathy; Cryo, cryoglobulinemic; eGFR, estimated glomerular filtration rate; FGN, fibrillary glomerulonephritis; GN, glomerulonephritis; Ig, immunoglobulins; IQR, interquartile range; LCPT, light chain proximal tubulopathy; MG, monoclonal gammopathy; MGRS, monoclonal gammopathy of renal significance; MIDD, monoclonal immunoglobulin deposition disease; *N*, number; ns, not significant; PCs, plasma cells; PGNMID, proliferative glomerulonephritis with monoclonal immunoglobulin deposits; sFLC, serum free‐light chains.

As expected, the median serum *M* protein level was low (40 mg/dL [IQR 0–323]), with no differences between the two groups (*p* = ns). Meanwhile, clonal identification in BM confirmed the presence of a small PC clone (8% [5–10]), similar in the two groups (*p* = ns). At histopathology assessment of the kidney, the other‐MGRS group consisted of renal lesions both with and without monoclonal immunoglobulin deposits, with the following distribution: monoclonal immunoglobulin deposition disease (MIDD) (39%, *n* = 7), light chain proximal tubulopathy (LCPT) (28%, *n* = 5), proliferative glomerulonephritis with monoclonal immunoglobulin deposits (PGNMID) (11%, *n* = 2), fibrillar glomerulonephritis (11%, *n* = 2), C3 glomerulopathy with monoclonal gammopathy (5.5%, *n* = 1), and cryoglobulinemic type 1 glomerulonephritis (5.5%, *n* = 1) (Table 1). Regarding nephrological parameters, renal impairment with eGFR < 60 mL/min was present in 28 patients (50%), being severe (eGFR < 30 mL/min) in 15 of them (27%), and 12 (21%) required dialytic treatment. Specifically, a higher percentage of patients in the AL amyloidosis group had eGFR ≥ 60 mL/min (63%, *n* = 25) than in the other‐MGRS group (19%, *n* = 3, *p* = 0.003) and the median serum creatinine was 1 (IQR 0.7–1.3) versus 2.3 mg/dL (IQR 1.4–3.5), respectively (*p* < 0.001). Proteinuria was mostly in the nephrotic range (median of 4 g per day in the whole population), higher in patients with AL amyloidosis (median of 4.5 g per day) than in the other‐MGRS subgroup (median of 2.4 g per day, *p* = 0.003).

### 3.2 Therapeutic Approaches, Responses, and Toxicity

All patients received a first‐line treatment, 22 (37%) required a second line of therapy and 16 (27%) additional therapies (see Table 2 for details). Frontline therapies consisted of bortezomib‐based regimens without autologous stem cell transplantation (ASCT) (*n* = 24, 40%), bortezomib‐based plus ASCT (*n* = 11, 18%), ASCT alone (*n* = 9, 15%), melphalan‐based (*n* = 13, 22%), and rituximab‐based (*n* = 3, 5%), with bortezomib being the most commonly used drug in all histologic groups. Twenty/60 patients (33%) underwent ASCT preceded or not by an induction phase with a bortezomib‐base regimen, with no significant differences among groups. Treatment regimens used at relapse are reported in Table S1; in this setting, PIs and IMiDs were the most used drugs.

**TABLE 2:** Treatment and hematological and renal responses.

	Overall, *N* = 60	AL amyloidosis‐MGRS, *N* = 42	Other‐MGRS, *N* = 18	*p* value
LOT, *N* (%)				
1 line	60 (100)	42 (100)	18 (100)	0.024
2 lines	22 (37)	19 (45)	3 (17)	0.084
≥ 3 lines	16 (27)	12 (29)	4 (22)	ns
First‐line therapy, *N* (%)				0.064
Bortezomib‐based w/o ASCT^a^	24 (40)	15 (36)	9 (50)	
Bortezomib‐based + ASCT^b^	11 (18)	6 (14)	5 (28)	
ASCT upfront	9 (15)	8 (19)	1 (5.6)	
Melphalan‐based	13 (22)	12 (29)	1 (5.6)	
Rituximab‐based	3 (5)	1 (2.4)	2 (11)	
Hematological response*, *N* (%)				
≥ VGPR	28 (56)	24 (67)	4 (28)	0.015
PR	15 (30)	7 (19)	8 (57)	0.016
SD	7 (14)	5 (14)	2 (14)	ns
Not evaluable	10	6	4	
Renal response**, *N* (%)				ns
Yes	32 (59)	22 (56)	10 (67)	
No	22 (41)	17 (44)	5 (33)	
Not evaluable	6	3	3	

Abbreviations: ASCT, autologous stem cell transplantation; CR, complete response; LOT, line of therapy; *N*, number; ns, not significant; PR, partial response; SD, stable disease; VGPR, very good partial response; w/o, without.

^a^ Bortezomib‐based regimens NOT followed by ASCT: bortezomib‐cyclophosphamide‐dexamethasone in 12 patients, bortezomib‐melphalan‐dexamethasone in 6 patients, and bortezomib‐dexamethasone in 6 patients.

^b^ Bortezomib‐based regimens followed by ASCT: bortezomib‐cyclophosphamide‐dexamethasone in 4 patients, bortezomib‐dexamethasone in 5 patients, and bortezomib‐thalidomide‐dexamethasone in 2 patients.

* Assessed according to International Myeloma Working Group (IMWG) criteria.

** Defined as a decrease of > 30% of 24 h proteinuria (in the absence of renal progression defined by a progressive decrease of > 25% of eGFR).

Hematologic response was evaluable in 50/60 patients (83%) (Table 2). The ORR was 86% (86% and 85% in the AL amyloidosis and other‐MGRS groups, respectively), with a median time to best response of 12 months (IQR 6–19). Particularly, 56% of patients achieved ≥ VGPR, more frequently in the AL amyloidosis group (67%, *n* = 24) than in the other MGRS group (28%, *n* = 4, *p* = 0.015). Notably, CR achievement was significantly correlated with ASCT, independently of prior bortezomib (*p* = 0.003) (Table S2).

Renal response assessment was available in 90% (*n* = 54) of the entire population. A renal response was obtained in 32 (59%) patients, and no significant differences were observed between the two subgroups analyzed (*p* = ns) (Table 2). Notably, three patients permanently discontinued dialysis, due to recovery of renal function. Of note, 21/28 (75%) patients with > VGPR and 15/18 (83%) patients with CR had a renal response (*p* = 0.049 and *p* = 0.026, respectively), not achieved by any patient with SD (*p* ≤ 0.001), showing a correlation between hematological and renal response (see Table S3), albeit 6 patients with a response ≥ VGPR had a renal progression. Conversely, there was no association between renal response and distinct renal histotypes at diagnosis (*p* = ns) or type of first‐line therapy (*p* = ns) (Tables S2 and S4, respectively).

Overall, the main toxicities related to first‐line therapy were hematological (in 25 patients [42%] of all grades and 23 patients [38%] of grade ≥ 3), infectious (in 14 patients [23%]; 5 [8%] of Grade ≥ 3), and neurological (peripheral neuropathy in 9 patients [15%]; 4 [7%] of grade ≥ 3). Two patients (3%), both with amyloidosis AL, died from systemic infection during first‐line bortezomib therapy, without achieving a hematologic response. Ten patients (16%) experienced other less frequent AEs (nausea, diarrhea, skin rash, hypertension, and hyperglycemia), Grade ≥ 3 in 3 patients (5%).

### 3.3 Survival Outcomes

At the time of the present analysis, 23 patients (38%) had progressed and 9 patients (15%) had died (17% [*n* = 7] in the AL amyloidosis group and 11% [*n* = 2] in the other‐MGRS group). Disease progression (44%, *n* = 4) and infections (22%, *n* = 2) were the most common causes of death, while one patient died from heart failure (AL amyloidosis group), one from stomach cancer (other MGRS group), and one from unknown reasons.

At a median follow‐up of 54.4 months (IQR 24.8–102.8), median PFS (mPFS) was 100.1 months (95% CI 34.9‐NR) and median OS was not reached (95% CI 129.8‐NR). No significant differences were observed between the two groups in PFS (HR = 1.15, 95% CI: 0.41–3.26, *p* = 0.79) and OS (HR = 0.42, 95% CI: 0.05–3.47, *p* = 0.423).

Univariate analysis of PFS in the whole population (Table 3), performed by patient main baseline characteristics, therapies, and responses, revealed that achieving ≥ VGPR hematological response after first‐line therapy was associated with improved PFS (HR = 0.21, 95% CI: 0.06–0.69, *p* = 0.010, mPFS, NR). In addition, baseline eGFR values showed a trend toward statistical significance (HR = 0.99, 95% CI: 0.97–1.00, *p* = 0.051). By contrast, the presence of cryoglobulinemic glomerulonephritis as a renal histotype had a negative impact on PFS (HR = 10.44, 95% CI: 1.19–91.51, *p* = 0.034; mPFS 8.903). Univariate analysis of PFS in the AL amyloidosis subgroup showed a consistent PFS benefit for patients with baseline eGFR ≥ 60 mL/min per 1.73 m^2^ (HR = 0.19, 95% CI: 0.05–0.77, *p* = 0.020; mPFS NR) and with hematological response ≥ VGPR (HR = 0.26, 95% CI: 0.07–0.98, *p* = 0.047; mPFS NR). Conversely, higher creatinine levels at baseline adversely affected PFS (HR = 1.44, 95% CI: 1.05–1.99, *p* = 0.025; mPFS NR). As for the other‐MGRS subgroup, no variables impacted PFS (Table 3).

**TABLE 3:** Univariate analysis for PFS by patient main baseline characteristics, therapies, and responses in the overall population and subgroups.

Univariate analysis	Overall				Amyloidosis AL				Other‐MGRS			
	*N*	mPFS (95% CI)	HR (95% CI)	*p*‐value	*N*	mPFS (95% CI)	HR (95% CI)	*p*‐value	*N*	mPFS (95% CI)	HR (95% CI)	*p*‐value
Age, years	59	NR	1.03 (0.98–1.09)	0.207	41	NR	1.06 (0.99–1.12)	0.086	18	56.90 (50.46,—)ᵇ	0.99 (0.91–1.07)	0.789
Serum M‐protein, mg/dL	43	NR	1.00 (0.99–1.00)	0.348	27	NR	0.99 (0.96–1.02)	0.407	16	56.90 (34.85,—)ᵇ	1.00 (1.00–1.00)	0.752
*K* sFLC, mg/L	60	NR	1.00 (1.00–1.00)	0.990	42	NR	1.00 (1.00–1.00)	0.441	18	56.90 (50.46,—)ᵇ	1.00 (1.00–1.00)	0.561
*λ* sFLC, mg/L	60	NR	1.00 (1.00–1.00)	**0.002**	42	NR	1.00 (1.00–1.00)	**0.003**	18	56.90 (50.46,—)ᵇ	0.96 (0.89–1.03)	0.212
Type of renal lesion	60								18			
AL		NR	—	—	—	NR	—	—	—	—	—	—
MIDD		56.90ᵃ	0.66 (0.09–5.06)	0.687						56.90ᵃ	0.08 (0.00–2.23)	0.138
LCPT		50.46ᵃ	0.88 (0.11–6.82)	0.902						50.46ᵃ	0.17 (0.01–3.76)	0.264
PGNMID		38.39^a^	1.57 (0.20–11.99)	0.666						38.39^a^	0.10 (0.00–3.70)	0.214
Monoclonal FGN		NR	0.00 (0.00‐Inf)	0.998						NR	0.00 (0.00‐Inf)	> 0.999
C3G		34.86^†^	3.66 (0.46–28.90)	0.219						34.86^†^	—	
Cryo GN		8.903^†^	10.44 (1.19–91.51)	**0.034**						8.903^†^		
Serum creatinine, mg/dL	57	NR	1.13 (0.86–1.48)	0.382	41	NR	1.44 (1.05–1.99)	**0.025**	16	56.90 (50.46,—)ᵇ	0.24 (0.04–1.59)	0.138
eGFR, mL/min	56		0.99 (0.97–1.00)	**0.051**	40		0.98 (0.96–0.99)	**0.006**	16		1.04 (0.99–1.11)	0.145
< 30 mL/min		NR	—			54.54 (2.168,—)ᵇ	—			NR	—	
≥ 60 mL/min		NR	0.38 (0.10–1.43)	0.154		NR	0.19 (0.05–0.77)	**0.020**		NR	1.19 (0.00‐Inf)	> 0.999
30–60 mL/min		22.00 (19.48,—)ᵇ	2.27 (0.73–7.04)	0.156		22.00 (20.70,—)ᵇ	0.93 (0.25–3.54)	0.921		34.97 (8.903,—)ᵇ	6,207,156,465 (0.00‐Inf)	> 0.999
24 h urine protein, g	57	NR	1.00 (1.00–1.00)	0.426	41	NR	1.00 (1.00–1.00)	0.729	16	56.90 (50.46,—)ᵇ	1.00 (1.00–1.00)	0.087
Dialysis, yes vs. no	56	56.90 (23.13,—)ᵇ	1.61 (0.59–4.38)	0.351	40	67.27 (20.44,—)ᵇ	1.78 (0.58–5.45)	0.315	16	56.90ᵃ	1.07 (0.11–10.57)	0.953
First‐line therapies	60				42				18			
ASCT upfront		NR	0.18 (0.02–1.51)	0.115		NR	0.00 (0.00‐Inf)	0.998		56.90^†^	1.52 (0.13–17.35)	0.737
Bortezomib‐based + ASCT		NR	0.77 (0.20–2.99)	0.704		NR	0.75 (0.14–3.87)	0.727		NR	1.46 (0.09–24.78)	0.794
Bortezomib‐based w/o ASCT		54.54 (34.86,—)ᵇ	—			54.54 (20.70,—)ᵇ	—			NR	—	
Melphalan‐based		41.59 (10.15,—)ᵇ	1.37 (0.46–4.08)	0.578		41.59 (10.15,—)ᵇ	1.25 (0.38–4.11)	0.717		NR		
Rituximab‐based		NR	1.80 (0.21–15.10)	0.589		NR	0.00 (0.00‐Inf)	> 0.999		11.65 (8.903,—)ᵇ	4,405,625,693 (0.00‐Inf)	> 0.999
ASCT, yes vs. no	56	NR	0.38 (0.12–1.18)	0.094	40	NR	0.26 (0.06–1.19)	0.082	16	NR	0.95 (0.15–5.95)	0.957
NO		NR	—	—	—	NR	—	—	—	50.46 (34.86,—)ᵇ	—	—
Hematological response					36				14			
CR	50		0.17 (0.04–0.76)	**0.021**		NR	0.22 (0.04–1.06)	0.059		NR	0.00 (0.00‐Inf)	> 0.999
(< CR)*		56.90 (50.33,—)ᵇ	—	—	—	NR	—	—	—	50.46 (19.48,—)ᵇ	—	—
≥ VGPR	50	NR	0.21 (0.06–0.69)	**0.010**	36	NR	0.26 (0.07–0.98)	**0.047**	14	NR	0.00 (0.00‐Inf)	> 0.999
(< VGPR)*		56.90 (20.44,—)ᵇ	—	—	—	NR	—	—	—	50.46 (19.48,—)ᵇ	—	—
Renal response	54	NR	0.42 (0.16–1.13)	0.085	39	NR	0.33 (0.10–1.12)	0.076	15	65.23 (50.46,—)ᵇ	0.79 (0.13–4.80)	0.800
(NO)*		56.90 (23.13,—)ᵇ	—	—	—	NR	—	—	—	56.90 (34.86,—)ᵇ	—	—

*Note:* *Reference level. ^(a) or (†)^ The reported median survival corresponds to the survival time of a single individual in subgroups where only one subject (†) or event (a) is present. ^(b)^ Upper confidence limit not estimable (NA) due to insufficient events beyond the median survival time. Values in bold refer to *p* value < 0.05, to emphasize statistical significance.

Abbreviations: ASCT, autologous stem cell transplantation; CI, confidence interval; C3G, complement 3 glomerulopathy; CR, complete response; Cryo, cryoglobulinemic; eGFR, estimated glomerular filtration rate; FGN, fibrillary glomerulonephritis; GN, glomerulonephritis; HR, hazard ratio; LCPT, light chain proximal tubulopathy; MGRS, monoclonal gammopathy of renal significance; MIDD, monoclonal immunoglobulin deposition disorder; mPFS, median PFS; NR, not reached; PFS, progression‐free survival; PGNMID, proliferative glomerulonephritis with monoclonal IgG deposits; PR, partial response; SD, stable disease; sFLC, serum free light chain; VGPR, very good partial response; w/o, without.

Multivariate analysis confirmed response ≥ VGPR, as an independent predictor of PFS in the entire population (≥ VGPR: HR = 0.29, 95% CI 0.09–0.84; *p* = 0.023; baseline eGFR: HR = 1.00, 95% CI: 0.99–1.02, *p* = 0.710) (Figure S1). Although the limitation of small numbers, in the AL amyloidosis group, response ≥ VGPR together with baseline eGFR ≥ 60 mL/min or between 30 and 60 mL/min compared to values below 30 mL/min per 1.73 m^2^ were identified as independent variables predicting improved PFS (≥ VGPR: HR 0.23; 95% CI: 0.06–0.82; *p* = 0.023; eGFR levels ≥ 60 mL/min: HR = 0.003; 95% CI 0.00–0.38; *p* = 0.018; eGFR levels 30‐60 mL/min: HR = 0.04, 95% CI: 0.001–0.94; *p* = 0.046; serum creatinine: HR = 0.11, 95% CI: 0.008–1.48, and *p* = 0.095) (Figure S2).

No variables were found to potentially influence OS, either in the general population or in each of the subgroups (Table S5).

## 4 Discussion

MGRS are relatively new and rare clinical entities, referred to as clonal hematologic disorders that produce a nephrotoxic monoclonal paraprotein, whose diagnosis is often challenging, leading to delays in treatment. Although the classification and characterization of MGRS have improved significantly in recent years, knowledge of this spectrum of disorders is still far from comprehensive and the optimal management of MGRS is still evolving. Currently, there is a lack of data on prognostic markers, hematologic/renal response criteria, risk of progression to symptomatic MM or other lymphoproliferative disease and outcomes, and treatment guidelines. To implement real‐life data, we retrospectively analyzed a multicenter cohort of 60 patients diagnosed with renal biopsy‐proven MGRS who had received mainly novel treatments.

Similarly to previous studies [8, 9, 26, 29], the most represented renal histopathological entity was AL amyloidosis, followed by the non‐organized deposits MIDD groups, while only a few had less recurrent types of MGRS such as C3 glomerulopathy with monoclonal gammopathy or cryoglobulinemic GN. Therefore, we compared clinical data between patients with AL amyloidosis and those with other rarer renal histopathologies.

As in other cases [20, 26], the analysis of demographics characteristics revealed a relatively young patient population, likely due to the requirement of renal biopsy to prove the diagnosis of MGRS and enrolment of patients receiving treatment, which are not always feasible in elderly patients. Likewise, the main laboratory and clinical characteristics described in our study are consistent with those in previous reports [9, 20, 26]. Particularly, hematologic evaluations conducted to identify the underlying clone showed in both groups only a small increase in plasma cells or B‐cells (by definition < 10%); correspondingly, the monoclonal immunoglobulin concentration and the sFLC levels were low, consistent with the presence of the typical “dangerous small” clone. Some laboratory features differed between AL and other‐MGRS groups. Indeed, we found that more patients in the AL amyloidosis group had a lambda monotypic light‐chain restriction and a higher median sFLC lambda compared with patients in the other‐MGRS group, supporting the importance of the sFLC assay for inferring clonality and the need for further research to evaluate its role as a potential prognostic and treatment response marker [29, 30].

Clinical presentations of MGRS are quite heterogeneous and depend on the affected segment of the nephron [31]. In our population, a renal impairment (eGFR < 60 mL/min) was present in half of the patients and about a third of patients experienced severe renal failure (eGFR < 30 mL/min), requiring dialytic treatment in 21% of cases. The comparison of nephrological parameters between the two subgroups showed that more patients in AL amyloidosis than those in the other‐MGRS group had a significantly better renal function, in terms of eGFR ≥ 60 mL/min and median serum creatinine. Instead, proteinuria in the nephrotic range and hypoalbuminemia were significantly elevated in patients with AL amyloidosis compared with those with other‐MGRS.

Currently, specific MGRS treatments directed against the deposited M‐protein are still lacking; therefore, clone‐directed therapy is the only feasible therapeutic option, aiming at blocking M‐protein production and preventing the progression of organ damage [23].

Treatment approaches in MGRS disorders remain heterogeneous due to a lack of data, particularly for MGRS subtypes other than AL amyloidosis. In our population, treatment choice was based on numerous factors, including age, comorbidities, performance status, drug availability, expected toxicity profile, and clinical experience of participating centers. Considering that the proteasome inhibitor bortezomib has proven to be a highly effective drug in the treatment of AL amyloidosis and MIDD while having a non‐renal metabolism [31, 32], it was the drug of choice in our cases, both first‐line and subsequent lines of therapy, for both subgroups. Overall, one‐third of patients underwent ASCT during the first line of therapy.

Hematological response criteria in MGRS disorders have not been established. Although response criteria for MM are used, these are not always adequate, as in 30%–40% of patients a malignant clone and/or abnormal laboratory values may not be identifiable [33, 34].

In our study, hematologic and renal response were not assessed in 17% and 10% of patients, respectively, and in the larger international retrospective study of Gozzetti et al. up to a quarter of patients had no hematologic and/or renal response evaluation, suggesting that there is room for improvement in the management of MGRS [26, 35]. We observed a high percentage of ORR and more than half of the patients had a renal response, defined according to the criteria established for AL amyloidosis [28]. A greater number of patients in AL amyloidosis than in the other‐MGRS groups achieved a high‐quality response equal to or superior to VGPR. Interestingly, of the 18 patients in CR, 11 received ASCT. However, there was no significant difference in the distribution of hematological responses across different types of treatments, likely due to the heterogeneity of the therapies used and the small number of patients in each subgroup. Instead, in line with some studies suggesting that the kidney response can be further improved by achieving deeper hematological responses [24, 26, 36, 37], our analysis revealed a significant correlation between hematological and renal responses. Indeed, a renal response was obtained by 75% of patients with hematological responses ≥ VGPR (83% in the case of CR) and by none of the patients with an SD. However, despite deep hematological responses, some patients still experienced a decline in kidney function over time. Conversely, neither the distinct renal histotypes at diagnosis nor the types of treatment, first‐line or subsequent lines, influenced the renal response.

The potential severity of MGRS disorders was underlined by 4 deaths due to disease progression, during a median follow‐up of approximately 4.5 years for the entire population. Survival analysis showed no significant difference between AL amyloidosis and the other‐MGRS group in terms of PFS and OS. Moreover, the achievement of a hematological response equal to VGPR, or better, was confirmed as the main independent predictor of prolonged PFS for the whole population and the subgroup of patients with AL amyloidosis. In addition, the presence of preserved renal function, with eGFR values at diagnosis between 30 and 60 mL/min and even above 60 mL/min per 1.73 m^2^, was identified as an independent variable predicting improved PFS in the AL amyloidosis group, although this result is limited by small numbers. No variables impacting PFS were identified in the other‐MGRS group, nor were any factors potentially influencing OS, either in the general population or in each of the subgroups.

These data on survival are in contrast with those reported by Gozzetti et al., which highlighted shorter OS in patients with AL amyloidosis than those with other‐MGRS and longer survival in patients with both hematologic and renal responses. However, this latter study did not clearly exclude patients with multiorgan amyloidosis, while we focused our analysis solely on patients with renal AL amyloidosis to guarantee consistency in the clinical presentation with the other MGRS histotypes.

To the best of our knowledge, our study has the longest follow‐up period so far reported. However, a non‐negligible proportion of patients received a melphalan‐based chemotherapeutic approach as the first line of therapy, whereas the current standard of care for patients with amyloidosis is based on the combination of the monoclonal antibody against CD38 daratumumab in addition to bortezomib and cyclophosphamide in many countries [21, 38, 39].

In conclusion, our retrospective patient population follows the typical manifestation frameworks of MGRS disorders in terms of clonal burden, laboratory, and clinical features. Although a greater number of patients with AL amyloidosis achieved a high‐quality response than other histological MGRS types, no significant difference in terms of survival outcomes emerged between the two subgroups. However, the high‐quality response was confirmed as the main independent predictor of prolonged PFS in all populations and in the AL amyloidosis group, in which even a better renal function at diagnosis was predictive of improved PFS. In addition, the achievement of a hematological response influenced the renal improvement after therapy.

More extensive research is warranted to support diagnosis and identify potential prognostic factors, with the goal of developing shared recommendations for early recognition, response evaluation, and proper management of MGRS. In this regard, the rarity and heterogeneity of the disease requires a collaborative approach, with national and international registries and the design of future prospective studies, aimed at improving knowledge about MGRS disorders.

## Author Contributions

**K. Mancuso:** conceptualization (equal), investigation (equal), project administration (equal), writing – original draft (lead), writing – review and editing (equal). **R. Mina:** investigation (equal), writing – review and editing (supporting). **S. Rocchi:** conceptualization (equal), investigation (equal), project administration (equal), writing – review and editing (equal). **E. Antonioli:** investigation (equal), writing – review and editing (supporting). **M. T. Petrucci:** investigation (equal), writing – review and editing (supporting). **F. Fazio:** investigation (equal), writing – review and editing (supporting). **A. Gozzetti:** investigation (equal), writing – review and editing (supporting). **M. Salvini:** investigation (equal), writing – review and editing (supporting). **C. S. Cartia:** investigation (equal), writing – review and editing (supporting). **G. Bertuglia:** investigation (equal), writing – review and editing (supporting). **F. Patriarca:** investigation (equal), writing – review and editing (supporting). **A. B. Dalla Palma:** investigation (equal), writing – review and editing (supporting). **S. Barbato:** project administration (equal), writing – original draft (equal), writing – review and editing (lead). **G. De Cicco:** investigation (equal), writing – review and editing (supporting). **F. Bigi:** investigation (equal), writing – review and editing (supporting). **E. Favero:** investigation (equal), writing – review and editing (supporting). **P. Tacchetti:** investigation (equal), writing – review and editing (supporting). **L. Pantani:** investigation (equal), writing – review and editing (supporting). **I. Rizzello:** investigation (equal), writing – review and editing (supporting). **M. Puppi:** investigation (equal), writing – review and editing (supporting). **M. Talarico:** investigation (equal), writing – review and editing (supporting). **V. Solli:** data curation (equal), formal analysis (equal), software (equal). **A. Kanapari:** formal analysis (equal). **M. Cavo:** conceptualization (equal), funding acquisition (equal), supervision (equal), writing – review and editing (equal). **E. Zamagni:** conceptualization (lead), funding acquisition (equal), investigation (equal), project administration (equal), supervision (equal), writing – review and editing (equal).

## Disclosure

KM has received honoraria from Celgene, Takeda, Amgen, Sanofi, and Janssen; RM has served on the advisory boards, speaker's bureau, and consultancy for Jannsen; SR has received honoraria from Amgen, Bristol‐Myers Squibb, GlaxoSmithKline, and Janssen; FP has served on the advisory boards and speaker bureau for Sanofi, GlaxoSmithKline, Amgen, Roche, Janssen, and Novartis; PT has received honoraria from Amgen, Bristol‐Myers Squibb/Celgene, Janssen, Takeda, AbbVie, Sanofi, GlaxoSmithKline, and Pfizer; LP has received honoraria from GlaxoSmithKline, Pfizer, and Sanofi; IR has received honoraria from Amgen, GlaxoSmithKline, and Sanofi and advisory role for GlaxoSmithKline; MC has served in a consulting/advisory role for Amgen, AbbVie, Bristol‐Myers Squibb, Celgene, GlaxoSmithKline, Janssen, Menarini Stemline, Sanofi, and Karyopharm Therapeutics, and has received honoraria from Amgen, AbbVie, Bristol‐Myers Squibb, Celgene, GlaxoSmithKline, Janssen, Menarini Stemline, Sanofi, and Karyopharm Therapeutics; EZ has received honoraria and has served in advisory role for Janssen, Bristol‐Myers Squibb, Sanofi, Amgen, GlaxoSmithKline, Pfizer, Oncopeptides, Menarini‐Stemline.

## Conflicts of Interest

The authors declare no conflicts of interest.

## Supporting information


Data S1.

## Data Availability

The data that support the findings of this study will be made available by the Corresponding Author, upon reasonable request.
