# Supplementary material for: Clinical Presentation and Long‐Term Survival Outcomes of Patients With Monoclonal Gammopathy of Renal Significance (MGRS): A Multicenter Retrospective Study
Source: Cancer Med. 2024 Nov 25;13(22):e70266. doi: 10.1002/cam4.70266 (PMC11588855; doi:10.1002/cam4.70266)
Supplement: Supplementary file 1 — Data S1. [file CAM4-13-e70266-s001.docx]

**SUPPLEMENTARY MATERIAL**

**Table S1. Subsequent therapeutic lines and relative hematological and renal responses**

|  | N | Overall | AL amyloidosis-MGRS | Other-MRGS | p-value |
| --- | --- | --- | --- | --- | --- |
| *Second line* | | | | | |
| Treatment, N (%)  ASCT  Bortezomib-based  IMIDs  Melphalan-based  Daratumumab  Rituximab  Other | 22 |  |  |  | 0.3 |
|  |  | 1 (4.5) | 1 (5.3) | 0 (0) |  |
|  |  | 12 (55) | 11 (58) | 1 (33) |  |
|  |  | 4 (18) | 3 (16) | 1 (33) |  |
|  |  | 1 (4.5) | 1 (5.3) | 0 (0) |  |
|  |  | 1 (4.5) | 1 (5.3) | 0 (0) |  |
|  |  | 1 (4.5) | 0 (0) | 1 (33) |  |
|  |  | 2 (9.1) | 2 (11) | 0 (0) |  |
| Hematological response*, N (%) | 18 |  |  |  | 0.5 |
| CR |  | 4 (22) | 4 (27) | 0 (0) |  |
| VGPR |  | 4 (22) | 4 (27) | 0 (0) |  |
| PR |  | 3 (17) | 2 (13) | 1 (33) |  |
| SD |  | 7 (39) | 5 (33) | 2 (67) |  |
| Renal response**, N (%) | 21 | 13 (62) | 12 (67) | 1 (33) | 0.5 |
| *Third line* | | | | | |
| Treatment, N (%) | 10 |  |  |  | 0.5 |
| Bortezomib-based |  | 2 (20) | 1 (13) | 1 (50) |  |
| IMIDs |  | 7 (70) | 6 (75) | 1 (50) |  |
| Melphalan-based |  | 1 (10) | 1 (13) | 0 (0) |  |
| Hematological response*, N (%) | 8 |  |  |  | 0.2 |
| CR |  | 2 (25) | 2 (33) | 0 (0) |  |
| VGPR |  | 3 (38) | 3 (50) | 0 (0) |  |
| PR |  | 1 (13) | 0 (0) | 1 (50) |  |
| SD |  | 2 (25) | 1 (17) | 1 (50) |  |
| Renal response**, N (%) | 8 | 4 (50) | 3 (43) | 1 (100) | >0.9 |
| *Fourth line* | | | | | |
| Treatment, N (%) | 5 |  |  |  | 0.4 |
| Bortezomib-based |  | 1 (20) | 0 (0) | 1 (5%) |  |
| IMIDs |  | 1 (20) | 0 (0) | 1 (50) |  |
| Daratumumab |  | 2 (40) | 2 (67) | 0 (0) |  |
| ASCT |  | 1 (20) | 1 (33) | 0 (0) |  |
| Hematological response*, N (%) | 4 |  |  |  | >0.9 |
| PR |  | 3 (7) | 2 (100) | 1 (50) |  |
| SD |  | 1 (2) | 0 (0) | 1 (50) |  |
| Renal response**, N (%) | 3 | 1 (33) | 1 (100) | 0 (0) | 0.3 |

*assessed according to IMWG criteria

**defined as a decrease of >30% of 24 h proteinuria (in the absence of renal progression defined by progressive decrease of >25% of eGFR)

**Abbreviations**: ASCT, autologous stem cell transplantation; CR, complete response; IMIDs, immunomodulatory drugs; N, number; PR, partial response; SD, stable disease; VGPR, very good partial response

**Table S2. Hematological and renal responses by upfront therapy**

|  | **N** | **Overall**  N = 60 | **ASCT ± bortezomib**  N=20 | **Bortezomib-based, no ASCT**  N = 24 | **Melphalan-based**  N = 13 | **Rituximab-based**  N = 3 | **p-value** |
| --- | --- | --- | --- | --- | --- | --- | --- |
| **Hematological response*, N (%)** | 50 |  |  |  |  |  |  |
| CR |  | 18 (36) | 11 (55) | 1 (5.9) | 6 (60) | 0 (0) | 0.003 |
| VGPR |  | 10 (20) | 3 (15) | 6 (35) | 1 (10) | 0 (0) | *ns* |
| PR |  | 15 (30) | 3 (15) | 9 (53) | 2 (20) | 1 (33) | *ns* |
| SD |  | 7 (14) | 3 (15) | 1 (5.9) | 1 (10) | 2 (67) | *ns* |
| ≥VGPR |  | 28 (56) | 14 (70) | 7 (41) | 7 (70) | 0 (0) | *ns* |
| **Renal response**, N (%)** | 54 | 32 (59) | 15 (75) | 9 (45) | 7 (64) | 1 (33) | *ns* |

*assessed according to IMWG criteria

**defined as a decrease of >30% of 24 h proteinuria (in the absence of renal progression defined by progressive decrease of >25% of eGFR)

**Abbreviations**: ASCT, autologous stem cell transplantation; CR, complete response; N, number; *ns*, not significant; PR, partial response; SD, stable disease; VGPR, very good partial response.

**Table S3. Correlation between hematological and renal responses to the first line of therapy in the overall population.**

| **Hematological response, N (%)** | **N** | **Overall**  **n=54** | **Renal response** | | | **p-value** |
| --- | --- | --- | --- | --- | --- | --- |
|  |  |  | **No**  **n=22** | | **Yes**  **n=32** |  |
| CR | 49 | 18 (37) | 3 (17) | 15 (48) | | 0.026 |
| VGPR | 49 | 10 (20) | 4 (22) | 6 (19) | | *ns* |
| PR | 49 | 14 (29) | 4 (22) | 10 (32) | | *ns* |
| SD | 49 | 7 (14) | 7 (39) | 0 (0) | | <0.001 |
| ≥VGPR | 49 | 28 (57) | 7 (39) | 21 (68) | | 0.049 |
| Unknown |  | 5 | 4 | 1 | |  |

**Abbreviations**: CR, complete response; N, number; *ns*, not significant; PR, partial response; SD, stable disease; VGPR, very good partial response.

**Table S4. Hematological and renal responses to the first line of therapy according to different renal histotypes**

|  | **N** | **Overall**  **N = 60** | **AL amyloidosis**  **N = 42** | **C3G with MG**  **N = 1** | **Cryo GN**  **N = 1** | **LCPT**  **N = 5** | **MIDD**  **N = 7** | **MFGN**  **N = 2** | **PGNMID**  **N = 2** | **p-value** |
| --- | --- | --- | --- | --- | --- | --- | --- | --- | --- | --- |
| **Hematological response, N (%)** | **50** |  |  |  |  |  |  |  |  | *ns* |
| CR |  | 18 (36) | 15 (42) | 0 (NA) | 0 (0) | 1 (25) | 2 (33) | 0 (0) | 0 (0) |  |
| VGPR |  | 10 (20) | 9 (25) | 0 (NA) | 0 (0) | 0 (0) | 1 (17) | 0 (0) | 0 (0) |  |
| PR |  | 15 (30) | 7 (19) | 0 (NA) | 1 (100) | 3 (75) | 2 (33) | 1 (50) | 1 (100) |  |
| SD |  | 7 (14) | 5 (14) | 0 (NA) | 0 (0) | 0 (0) | 1 (17) | 1 (50) | 0 (0) |  |
| ≥VGPR |  | 28 (56) | 24 (67) | 0 (NA) | 0 (0) | 1 (25) | 3 (50) | 0 (0) | 0 (0) | *ns* |
| *Unknown* |  | 10 | 6 | 1 | 0 | 1 | 1 | 0 | 1 |  |
| **Renal response, N (%)** | 54 | 32 (59) | 22 (56) | 0 (0) | 1 (100) | 2 (67) | 4 (67) | 1 (50) | 2 (100) | *ns* |
| *Unknown* |  | 6 | 3 | 0 | 0 | 2 | 1 | 0 | 0 |  |

**Abbreviations:** C3G, complement 3 glomerulopathy; CR, complete response; Cryo, cryoglobulinemic; GN, glomerulonephritis; LCPT, light chain proximal tubulopathy; MFGN, monoclonal fibrillary glomerulonephritis; MG, monoclonal gammopathies; MIDD, monoclonal immunoglobulin deposition disease; N, number; *ns*, not significant; PGNMID, proliferative glomerulonephritis with monoclonal immunoglobulin deposits; PR, partial response; SD, stable disease; VGPR, very good partial response.

**Table S5. Univariate analysis for OS, by patient main baseline characteristics, therapies, and responses in the overall population and in different subgroups**

| **Univariate Analysis in OS** | **Overall** | | | | **Amyloidosis AL** | | | | **Other-MGRS** | | | |
| --- | --- | --- | --- | --- | --- | --- | --- | --- | --- | --- | --- | --- |
|  | **N** | **HR** | **95% CI** | **p-value** | **N** | **HR** | **95% CI** | **p-value** | **N** | **HR** | **95% CI** | **p-value** |
| **Age**, yrs | 59 | 1.09 | 1.01-1.19 | 0.035 | 41 | 1.08 | 0.99-1.17 | 0.082 | 18 | 1.21 | 0.81-1.80 | 0.348 |
| **Serum M-protein**, mg/dL | 43 | 1.00 | 0.99-1.01 | 0.445 | 27 | 0.99 | 0.97-1.02 | 0.518 | 16 | 1.00 | 0.99-1.01 | 0.966 |
| **K sFLC**, mg/L | 60 | 1.00 | 1.00-1.00 | 0.544 | 42 | 1.00 | 1.00-1.00 | 0.752 | 18 | 1.00 | 0.98-1.01 | 0.581 |
| **λ sFLC**, mg/L | 60 | 1.00 | 1.00-1.00 | 0.318 | 42 | 1.00 | 1.00-1.00 | 0.403 | 18 | 1.00 | 0.88-1.14 | 0.992 |
| **Type of renal lesion** | 60 |  |  |  |  |  |  |  | 18 |  |  |  |
| MIDD |  | 0.00 | 0.00-Inf | 0.999 |  |  |  |  |  | 1.00 | 0.00-Inf | >0.999 |
| LCPT |  | 0.00 | 0.00-Inf | 0.999 |  |  |  |  |  | 1.00 | 0.00-Inf | >0.999 |
| PGNMID |  | 3.84 | 0.46-32.13 | 0.215 |  |  |  |  |  | 16,537,034,053 | 0.00-Inf | >0.999 |
| Monoclonal FGN |  | 0.00 | 0.00-Inf | >0.999 |  |  |  |  |  | 1.00 | 0.00-Inf | >0.999 |
| C3G |  | 0.00 | 0.00-Inf | >0.999 |  |  |  |  |  | — | — |  |
| Cryo GN |  | 0.00 | 0.00-Inf | >0.999 |  |  |  |  |  |  |  |  |
| **Serum creatinine**, mg/dL | 57 | 0.99 | 0.64-1.55 | 0.987 | 41 | 1.26 | 0.81-1.96 | 0.313 | 16 | 0.000 | 0.00-Inf | 0.997 |
| **eGFR**, ml/min | 56 | 0.99 | 0.97-1.02 | 0.582 | 40 | 0.98 | 0.96-1.01 | 0.167 | 16 | 1.16 | 0.82-1.64 | 0.401 |
| <30ml/min |  | — | — |  |  | — | — |  |  | — | — |  |
| ≥60 ml/min |  | 1.62 | 0.17-15.58 | 0.678 |  | 0.75 | 0.08-7.18 | 0.800 |  |  |  |  |
| 30-60 ml/min |  | 5.32 | 0.59-47.90 | 0.136 |  | 2.68 | 0.27-26.29 | 0.398 |  | 5,682,308,411 | 0.00-Inf | >0.999 |
| **24h urine protein** | 57 | 1.00 | 1.00-1.00 | 0.098 | 41 | 1.00 | 1.00-1.00 | 0.181 | 16 | 1.00 | 1.00-1.00 | 0.370 |
| **Dialysis**, yes vs no | 56 | 0.99 | 0.20-4.95 | 0.994 | 40 | 1.10 | 0.21-5.68 | 0.911 | 16 | 0.00 | 0.00-Inf | >0.999 |
| **First-lines therapies** | 60 |  |  |  | 42 |  |  |  | 18 |  |  |  |
| ASCT upfront |  | 0.00 | 0.00-Inf | 0.999 |  | 0.00 | 0.00-Inf | 0.999 |  | 1.00 | 0.00-Inf | >0.999 |
| Bortezomib-based + ASCT |  | 1.83 | 0.39-9.148 | 0.459 |  | 1.37 | 0.23-8.24 | 0.728 |  | 1,966,487,631 | 0.00-Inf | >0.999 |
| Bortezomib-based w/o ASCT |  | — | — |  |  | — | — |  |  | — | — |  |
| Melphalan-based |  | 0.92 | 0.15-5.51 | 0.925 |  | 0.59 | 0.10-3.57 | 0.567 |  |  |  |  |
| Rituximab-based |  | 0.00 | 0.00-Inf | >0.999 |  | 0.00 | 0.00-Inf | >0.999 |  |  |  |  |
| **ASCT**, yes vs no | 56 | 1.09 | 0.24-4.90 | 0.907 | 40 | 0.77 | 0.14-4.23 | 0.765 | 16 | 1,363,379,458 | 0.00-Inf | >0.999 |
| **Hematological response ≥VGPR** | 50 | 0.29 | 0.05-1.56 | 0.148 | 36 | 0.28 | 0.05-1.69 | 0.164 | 14 | 0.00 | 0.00-Inf | >0.999 |
| **Renal response** | 54 | 0.50 | 0.12-2.00 | 0.323 | 39 | 0.36 | 0.08-1.63 | 0.183 | 15 | 1,027,621,176 | 0.00-Inf | >0.999 |

*Median OS was not reached for any of the variables listed in the table either in the overall population or in the subgroups.*

**Abbreviations:** ASCT, autologous stem cell transplantation; C3G, complement 3 glomerulopathy; CI, confidence interval; Cryo, cryoglobulinemic; eGFR, estimated glomerular filtration rate; FGN, fibrillary glomerulonephritis; GN, glomerulonephritis; HR, hazard ratio; LCPT, light chain proximal tubulopathy; MGRS, monoclonal gammopathy of renal significance; MIDD, monoclonal immunoglobulin deposition disorder; PFS, progression-free survival; PGNMID, proliferative glomerulonephritis with monoclonal IgG deposits; sFLC, serum free light chain; VGPR, very good partial response; w/o, without; yrs, years.

**Figure S1. Multivariate analysis for PFS in the overall population**


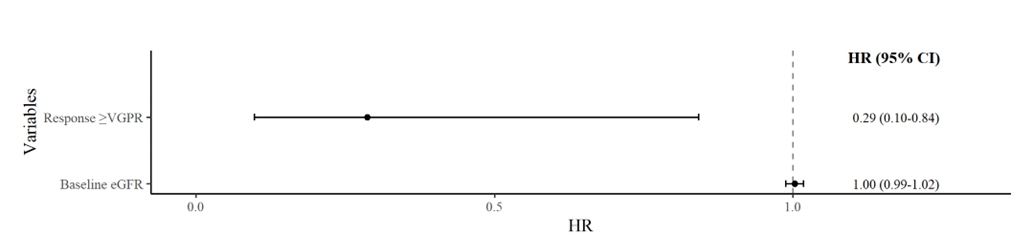


Events. 18, Global p-value (Log-Rank). 0.039750475

AIC. 104.08, Concordance Index. 0.69

**Abbreviations**: CI, confidence interval; eGFR, estimated glomerular filtration rate; HR, hazard ratio; VGPR, very good partial response.

**Figure S2. Multivariate analysis for PFS in the subgroup of patients with AL amyloidosis-MGRS**


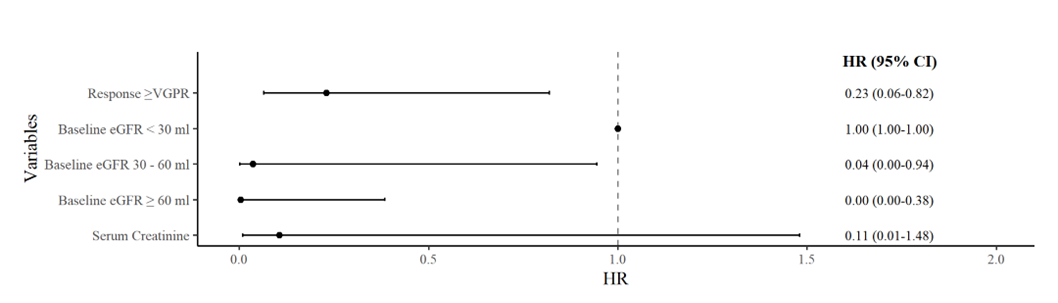


Events. 13, Global p-value (Log-Rank). 0.0068727731

AIC. 68.36, Concordance Index. 0.77

**Abbreviations**: CI, confidence interval; eGFR, estimated glomerular filtration rate; HR, hazard ratio; VGPR, very good partial response.
